# The Lung Elastin Matrix Undergoes Rapid Degradation Upon Adult Loss of *Hox5* Function

**DOI:** 10.3389/fcell.2021.767454

**Published:** 2021-11-26

**Authors:** Mu-Hang Li, Leilani M. Marty-Santos, Paul R. van Ginkel, Aubrey E. McDermott, Andrew J. Rasky, Nicholas W. Lukacs, Deneen M. Wellik

**Affiliations:** ^1^Genetics Training Program, University of Wisconsin-Madison, Madison, WI, United States; ^2^Department of Cell and Regenerative Biology, University of Wisconsin School of Medicine and Public Health, Madison, WI, United States; ^3^Department of Pathology, University of Michigan, Ann Arbor, MI, United States

**Keywords:** *Hox* genes, lung homeostasis, extracellular matrix, distal lung fibroblasts, lung macrophages, brochopulmonary dysplasia, emphysema

## Abstract

*Hox* genes encode transcription factors that are critical for embryonic skeletal patterning and organogenesis. The *Hoxa5*, *Hoxb5*, and *Hoxc5* paralogs are expressed in the lung mesenchyme and function redundantly during embryonic lung development. Conditional loss-of-function of these genes during postnatal stages leads to severe defects in alveologenesis, specifically in the generation of the elastin network, and animals display bronchopulmonary dysplasia (BPD) or BPD-like phenotype. Here we show the surprising results that mesenchyme-specific loss of *Hox5* function at adult stages leads to rapid disruption of the mature elastin matrix, alveolar enlargement, and an emphysema-like phenotype. As the elastin matrix of the lung is considered highly stable, adult disruption of the matrix was not predicted. Just 2 weeks after deletion, adult *Hox5* mutant animals show significant increases in alveolar space and changes in pulmonary function, including reduced elastance and increased compliance. Examination of the extracellular matrix (ECM) of adult *Tbx4rtTA; TetOCre; Hox5a*^f^*a*^f^*bbcc* lungs demonstrates a disruption of the elastin network although the underlying fibronectin, interstitial collagen and basement membrane appear unaffected. An influx of macrophages and increased matrix metalloproteinase 12 (MMP12) are observed in the distal lung 3 days after *Hox5* deletion. In culture, fibroblasts from *Hox5* mutant lungs exhibit reduced adhesion. These findings establish a novel role for *Hox5* transcription factors as critical regulators of lung fibroblasts at adult homeostasis.

## Introduction

*Hox* genes encode transcription factors that are well known for their role in patterning the anterior-posterior (AP) body axis during embryogenesis. All mammals have a total of 39 *Hox* genes located in four, tightly linked chromosomal clusters, subdivided into 13 related paralogous groups based on their expression and shared function. In addition to their roles in patterning the skeleton, *Hox* paralog groups also function redundantly in the proper formation of many organs, including the thymus, thyroid, lungs, pancreas, kidney and reproductive tract (Jeannotte et al., 1993; Aubin et al., 1997; Taylor et al., 1997; Manley and Capecchi, 1998; Wellik et al., 2002; Yallowitz et al., 2011). Multiple studies have demonstrated the functional redundancy exhibited by the members of paralog groups and loss-of-function of multiple paralogous genes results in more severe phenotypes than loss of a single *Hox* gene (Chisaka and Capecchi, 1991; Horan et al., 1995; Chen and Capecchi, 1997, 1999; Manley and Capecchi, 1998; Wellik et al., 2002, p. 11; Wellik and Capecchi, 2003; McIntyre et al., 2007; Boucherat et al., 2013; Hrycaj et al., 2015; Larsen et al., 2015).

Developmentally, critical roles for *Hox5* genes have been demonstrated in skeleton patterning, central nervous system formation, and lung organogenesis (Tuggle et al., 1990; Jeannotte et al., 1993; Aubin et al., 1997; Mandeville et al., 2006; McIntyre et al., 2007; Hrycaj et al., 2015, 2018a). During lung development, *Hox5* genes (*Hoxa5*, *Hoxb5*, and *Hoxc5*) are exclusively expressed in the mesenchyme of the lung (Aubin et al., 1997; Boucherat et al., 2013; Hrycaj et al., 2015). *Hoxa5* single homozygous mutants (*Hox5 aaBBCC*) exhibit a reduction in *Ttf-1* and *Hnf-3* expression and defects in surfactant production. There is also a high rate of perinatal lethality associated with improper tracheal morphogenesis and occlusion of the proximal airways. *Hoxb5* and *Hoxc5* single-mutant mice exhibit no overt embryonic lung phenotypes and are viable, as are *Hoxb5/Hoxc5* double mutant animals (Boucherat et al., 2013). The extent of functional redundancy of all three *Hox5* alleles was demonstrated by generating *Hoxa5*; *Hoxb5*; *Hoxc5* triple mutant embryos *(Hox5 aabbcc)*. Lungs from these embryos undergo only a few early branches and newborn animals die with severely hypoplastic lungs due to the loss of Wnt2/2b signaling in the early lung mesoderm. Compound, 4-allele *Hox5* mutant (*Hox5 AabbCc*) lungs show no observable defect at embryonic stages, but display expanded, simplified alveoli at postnatal stages compared to controls (Hrycaj et al., 2015). Expression of all three *Hox5* genes in the lung decreases from mid to late embryogenesis, then peaks to its highest level at postnatal stages, but is maintained throughout adult life. Postnatal deletion of *Hoxa5* in the background of *Hoxb5/Hoxc5* nulls leads to BPD (Hrycaj et al., 2018a).

In this study, we demonstrate that *Hox5* function remains important at adult stages for proper lung homeostasis. When *Hoxa5* deletion is induced at adult stages, the elastin matrix is disrupted within days after deletion. The distal airways expand and pulmonary function tests demonstrate that mutant lungs become significantly more compliant and less elastic just 2-weeks after deletion. The matrix disruption appears to be specific to elastin as laminin, interstitial collagen and fibronectin scaffolds appear unaffected. Examination of the distal lung just 3 days post-deletion shows increased expression of matrix metalloproteinase 12 (MMP12), also known as neutrophil elastase, and an influx of F4/80 + and CD68 + macrophage populations. Similar to what we previously reported at postnatal stages, fibroblasts from the *Hox5* triple, adult conditional mutant lung exhibit reduced adhesion and decreased integrin α5 protein expression. Our results are consistent with a model in which induced loss of fibroblast cell adhesion leads to elastin matrix instability. This work demonstrates that lung maintenance requires continued *Hox5* function in lung fibroblasts. Our work provides insight into the pathophysiological process and putative targets for molecular and cellular therapies for lung diseases.

## Materials and Methods

### Mice and Tissue Isolation

All mice used in this study have been previously reported (Zhang et al., 2013; Hrycaj et al., 2018a). Mice were treated with 2 mg/ml Doxycycline (DOT Sci., #DSD43020) (in water with 2.5 mg sucrose added per ml) at the age of 8 weeks for 3 days or 2 weeks. Mice were euthanized and perfused with phosphate buffered saline (PBS) via the right ventricle. Lungs were isolated, inflated and fixed as previously reported (Hrycaj et al., 2018b). The left lung lobes were vacuum embedded in paraffin; the right superior lung lobes were embedded in OCT (Fisher Sci., #23730571); the right middle and inferior lobes were digested for fibroblast isolation; the right accessory lobes were used for protein or RNA extraction. All experiments were performed following protocols approved by the Institutional Animal Care and Use Committee (IACUC) guidelines at the University of Michigan or the University of Wisconsin-Madison.

### Lung Whole Mount Imaging

Adult left lung lobes were fixed in 4% paraformaldehyde (PFA) in PBS overnight at 4°C then transferred to absolute MeOH through MeOH/PBS dilution series: 25, 50, 75 and 100% MeOH. Tissues then were incubated in Dent’s bleach (MeOH:DMSO:30% H_2_O_2_ = 4:1:1) for 2 h at room temperature to remove any coloration, and transferred to absolute MeOH for imaging on a Leica MZ125 dissecting microscope. PFA (Sigma-Aldrich, #P6148), MeOH (Sigma-Aldrich, #179337), DMSO (Sigma-Aldrich, #D2650), H_2_O_2_ (Sigma-Aldrich, #323381).

### Chord Length Analyses

Hematoxylin (Fisher Sci., #SH30500D) and Eosin (Fisher Sci., #SE23) staining was performed on 7 μm paraffin lung sections. Images were captured on a Nikon Ds-Fi3 camera. Mean alveolar chord length (MACL) measurements were taken using the grid function on ImageJ 2.0, as previously described (Sajjan et al., 2009).

### Pulmonary Function Tests

Analyses were performed as previously described (Hrycaj et al., 2018b). Briefly, 10 week old control and *Tbx4rtTA; TetOCre; Hox5a*^f^*a*^f^*bbcc* mice (treated with Dox from 8 to 10 weeks) were anesthetized prior to the insertion of a tracheal tube. Mechanical breathing measurements were performed at baseline to examine changes in lung function.

### Immunohistochemistry/Immunofluorescence

Paraffin sections were deparaffinized in xylenes and rehydrated in an ethanol series prior to antigen retrieval in 10 mM Sodium Citrate buffer. Cryosections were washed in PBS to remove excess OCT. Sections were blocked in 5% Normal Donkey Serum (Sigma-Aldrich, #566460) and incubated in primary antibodies in 4*^o^*C overnight. A complete list of primary antibodies and the dilution used is provided in Supplementary Table 1. Sections were rinsed, incubated in secondary antibodies at room temperature for 2 h, with a 10 min 1 μg/ml DAPI (Thermo Sci., #62248) incubation at room temperature and mounted using ProLong Gold mountant (Fisher Sci., #P36930). Images were captured on a Nikon Eclipse Ti-U camera or on a Keyence BZ-X810 fluorescence microscope.

### RNA Extraction and Quantitative Reverse Transcription PCR

Total RNA was extracted from the right accessory lobe of wildtype and mutant mice using the RNeasy Mini Kit (Qiagen, #74104) and dissolved in 32 μl of DNase/RNase-free deionized water. cDNA was synthesized from 1 μg RNA using the iScript Reverse Transcription Supermix (Bio-Rad, #1708841). Quantitative real-time PCR was conducted using 2 × SYBR Green qPCR Master Mix (Fisher Sci., #4309155) on a StepOnePlus™ Real-Time PCR System Machine (Fisher Sci., #4376600). Threshold cycles (Ct) in target gene expression were calculated and compared to Ct values of house-keeping gene β*-actin*. Primers for quantitative reverse transcription PCR (RT-qPCR) are listed in Supplementary Table 2.

### Western Blot Analysis

Lung right accessory lobes were lysed in radioimmunoprecipitation assay buffer (50 mM Tris⋅HCl, pH 7.2, 150 mM NaCl, 0.1% Triton X-100, 1% sodium deoxycholate, 5 mM EDTA) containing Complete Mini Protease Inhibitor Mixture (Sigma-Aldrich, #11873580001), and extracts were cleared by centrifugation at 20,000× *g* for 30 min at 4°C. Total protein content was assessed using the Pierce BCA protein assay kit (Thermo Sci. #23227) and analyzed by SDS/PAGE after boiling in Laemmli sample buffer (Bio-Rad, #1610737). Proteins were transferred to low-fluorescence polyvinylidene fluoride (Cytiva, #10600022), blocked in 3% BSA with sodium azide, and probed with primary antibodies as indicated in Supplementary Table 1. Fluorescence signals were detected on an Azure imaging system or a LI-COR Odyssey Fc imaging system. Western blot densitometry analysis was quantified using ImageJ 2.0.

### Lung Fibroblast Isolation and Primary Culture

Following perfusion with PBS and lung isolation, the trachea and proximal airway were removed, and the right middle and right inferior lung lobes were minced and digested in 2 mg/ml Collagenase I (Gibco, #17100017) and 3 mg/ml Dispase (Gibco, #17105041) for 2 h at 37°C. This cell suspension was incubated with red blood cell lysis buffer on ice and filtered through a 100 μm nylon cell strainer (Fisherbrand, #22363549). Cells were then washed twice in fresh DMEM/F12 media (Gibco, #11320033). Cells were plated in 6-well tissue culture plates using DMEM/F12 media supplemented with 10% FBS (Gibco, #10437010) and 1% 10,000 U/ml Penicillin-Streptomycin (Gibco, #15140122). Media was changed every third day.

### Adhesion Assay

Assays were performed as previously described (Hrycaj et al., 2018a). Non-tissue culture treated polystyrene 96-well flat bottom microtiter plates (Denville, # T1097) were coated and incubated with bovine plasma fibronectin (Sigma-Aldrich, #F1141) at 20 μg/ml for 1 h at 37°C. Plates were then blocked with 100 μl/well of 1% BSA (Sigma-Aldrich, #A7906) in serum-free DMEM/F12 (Gibco, #11320033) for 30 min at 37°C. Fibroblasts were seeded at 10,000 cells per individual well. Plates were centrifuged (top side up) at 10 × g for 5 min to reduce the variability inherent in the settling of cells onto the plate surface and were incubated for 1 h at 37°C with 5% CO_2_. Non-adherent cells were removed by centrifugation (top side down) at 48 × g for 5 min. Adherent cells remaining on the plate were fixed and stained with 1% formaldehyde (Fisher Sci., #BP228-100), 0.5% crystal violet (Sigma-Aldrich, #C6158), 20% MeOH followed by PBS washes. Individual wells were imaged on a Leica MZ125 dissecting microscope and manually quantified using ImageJ 2.0 cell counter function.

### Statistical Analyses

GraphPad Prism software (version 8.4.3 for macOS) was used to perform unpaired Student’s *t*-test analysis, with *P*-values less than 0.05 considered significant and *P*-values greater than or equal to 0.05 considered not significant.

## Results

### Conditional *Hox5* Triple Mutant Adult Mice Exhibit Expanded Distal Airspaces and Altered Lung Mechanics

**FIGURE 1:**
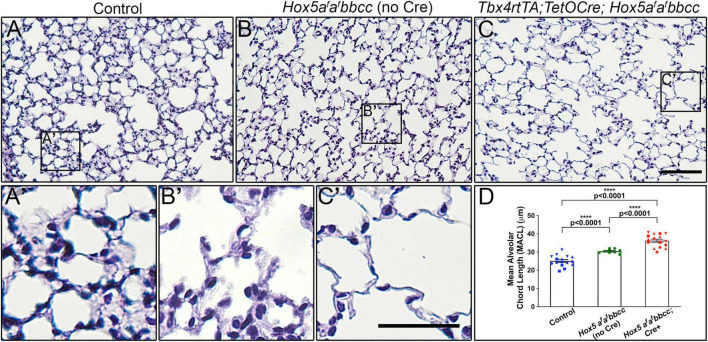
Deletion of *Hox5* function at adult stages leads to distal lung airway expansion. H&E sections through 10-week-old wild-type animals **(A,A’)**, *Hox5a*^*f*^a^*f*^*bbcc* mutants (no Cre) **(B,B’)** and *Hoxa5*^*LoxP/LoxP*^; *Hoxb5*^*– /–*^; *Hoxc5*^*– /–*^; *Tbx4rtTA; TetOCre* + mutants (*Hox5a*^*f*^a^*f*^*bbcc; Cre* +, **C,C’**), treated with Doxycycline from 8 to 10 weeks of age. Measured mean alveolar chord length (MACL) values **(D)**. *Hox5a*^*f*^a^*f*^*bbcc* double mutant animals (*Hoxa5* floxed alleles with no Cre) exhibit enlarged, simplified alveoli with a ∼20% increase in alveolar chord length compared to control lungs. The distal airway expansion phenotype with adult, conditional *Hox5* triple mutants increases by ∼45% compared to control lungs. Each shape represents an individual animal. Control, *Hox5AABBCC*. Scale bars: 50 μm **(C)**; 25 μm **(C’)**. *P*-values and statistical significance (****P < 0.0001) were determined by an unpaired Student’s *t*-test.

To investigate whether *Hox5* plays a role in the adult lung, we used our previously described *Hoxa5* conditional allele combined with the lung mesenchyme-specific *Tbx4rtTA*; *TetOCre* driver and enacted deletion with Doxycycline (Dox) beginning at 8 weeks of age in the presence of *Hoxb5/Hoxc5* loss-of-function (*Hoxa5*^LoxP/LoxP^*; Hoxb5^–/–^; Hoxc5^–/–^; Tbx4rtTA; TetOCre* +) (referred to as *Hox5* adult conditional mutants or as *Tbx4rtTA; TetOCre; Hox5a*^f^*a*^f^*bbcc* throughout) (Hrycaj et al., 2018a). Examination of the lungs 2 weeks after the initiation of *Hox5* deletion (at 10 weeks of age) resulted in significantly expanded distal airspaces that are clearly visible in the whole mount lung tissue after inflation (Supplementary Figure 1). This is supported by histological examination of sections and morphological measurements of mean alveolar chord length (MACL). *Tbx4rtTA; TetOCre; Hox5a*^f^*a*^f^*bbcc* Dox-treated mutant animals demonstrate a ∼45% increase in MACL 2-weeks post-deletion and thinned septal walls compared to wild-type animals (Figures 1A,A’,C,C’,D). Additionally, adult mice carrying only the *Hox5bbcc* alleles in the absence of Cre only developed a moderate phenotype (∼20% increase in MACL) (Figures 1B,B’,D), demonstrating that conditional, loss of *Hoxa5* function contributes directly to the adult phenotype.

**FIGURE 2:**
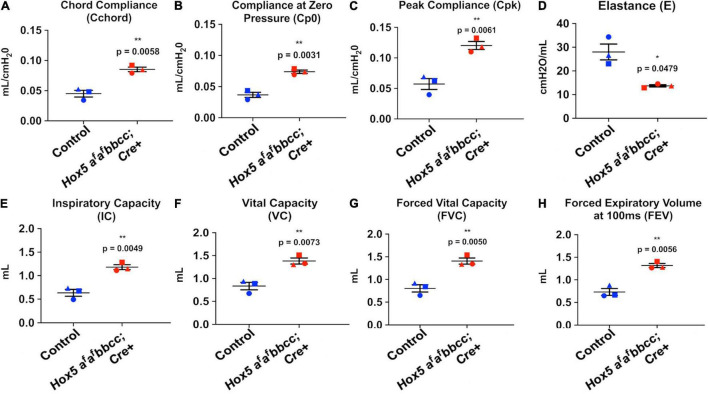
Adult conditional *Hox5* mutant lungs exhibit abnormal pulmonary function. Lung compliance, elastance and volume were measured in mice by fast flow maneuvers using orotracheal intubation and tracheostomy. Increased lung compliance of Dox-treated (8–10 weeks) *Hox5* conditional mutants is indicated by significant increases in chord compliance (Cchord) **(A)**, compliance at zero pressure (Cp0) **(B)**, and peak compliance (Cpk) **(C)**. *Hox5* adult conditional mutants also exhibit a significant decrease in elastance (E) **(D)**, and a significant increased lung volume indicated in inspiratory capacity (IC) **(E)**, vital capacity (VC) **(F)**, forced vital capacity (FVC) **(G)**, and forced expiratory volume (FEV) **(H)**. Each shape represents an individual animal. *P*-values and statistical significance (*0.01 ≤ *P* < 0.05; **0.001 ≤ *P* < 0.01) were determined by an unpaired Student’s *t*-test.

Measurements of pulmonary function revealed changes in the *Hox5* adult 10-week-old conditional mutants (Dox treated from 8 to 10 weeks) consistent with an emphysema-like phenotype. Lung compliance was increased as measured by increased chord compliance (Cchord), compliance at zero pressure (Cp0) and peak compliance (Cpk), with changes accompanied by a significant decrease in elastance (Figures 2A–D). Concomitantly, *Hox5* adult conditional mutants exhibit increased lung volumes, demonstrated by significant increases in inspiratory capacity (IC), vital capacity (VC), forced vital capacity (FVC), and forced expiratory volume (FEV) (Figures 2E–H).

### The Elastin Network Is Disrupted in *Hox5* Mutant Lungs, but Cell Types and Other Extracellular Matrix Components Appear Normal

**FIGURE 3:**
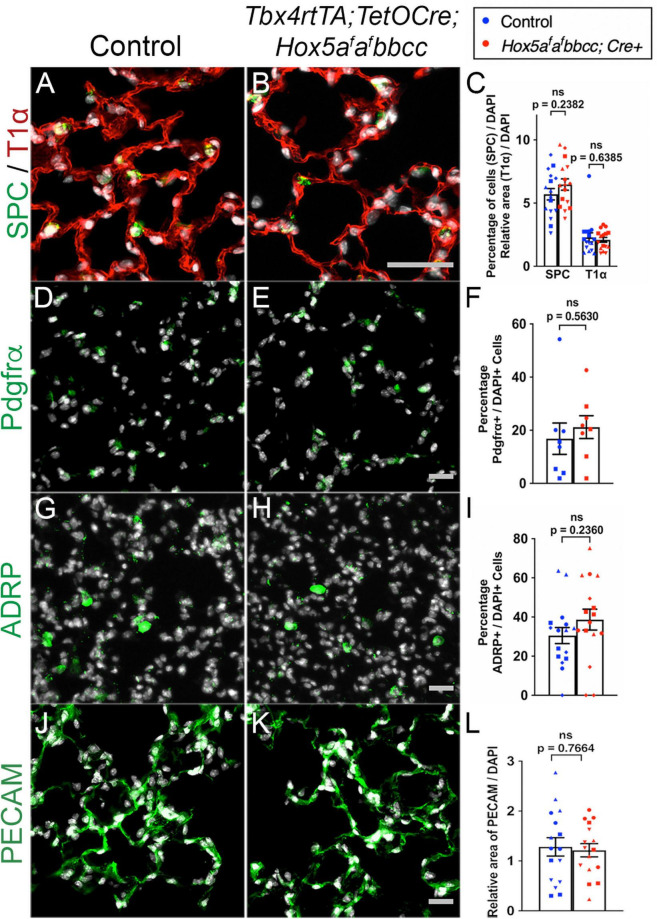
Distal lung cell types are present and appear similar in controls and *Hox5* conditional mutants. Lung paraffin sections **(A,B,G,H,J,K)** and cryosections **(D,E)** of control and *Hox5* adult, conditional mutant mice show similar expression of SPC (green, AECII cells), T1α (red, AECI cells), PDGFRα (green, fibroblasts), ADRP (green, lipofibroblasts) and PECAM (green, endothelial cells); DAPI in gray. Quantification of pixel intensity of T1α **(C)**, and PECAM **(L)** were normalized to pixel intensity of DAPI per field image. Quantifications of SPC **(C)**, PDGFRα **(F)** and ADRP **(I)** cell numbers were normalized to DAPI-positive cell numbers in each panel quantified. Each shape represents an individual animal (ns, not significant). Scale bars: 50 μm. *P*-values were determined by an unpaired Student’s *t*-test.

To examine whether changes occurred in distal lung cell composition, we examined epithelial, fibroblast, and endothelial cell populations in the distal lung after mesenchymal deletion of *Hoxa5*. We observed no significant changes in the morphology or distribution in T1α + alveolar epithelial type I cells (AECI) or Surfactant Protein C (SPC) + alveolar epithelial type II cells (AECII) (Figures 3A–C). We also observed no changes in the number or relative distribution of Platelet-derived growth factor receptor alpha (PDGFRα) + fibroblasts (Figures 3D–F), Adipocyte differentiation-related protein (ADRP) + lipofibroblasts (Figures 3G–I), or Platelet endothelial cell adhesion molecule (PECAM) + endothelial cells (Figures 3J,K) in the distal lung of the *Hox5* mutants. Additionally, no changes in mRNA levels of these cell markers was observed using RT-qPCR (Supplementary Figures 2A–E).

**FIGURE 4:**
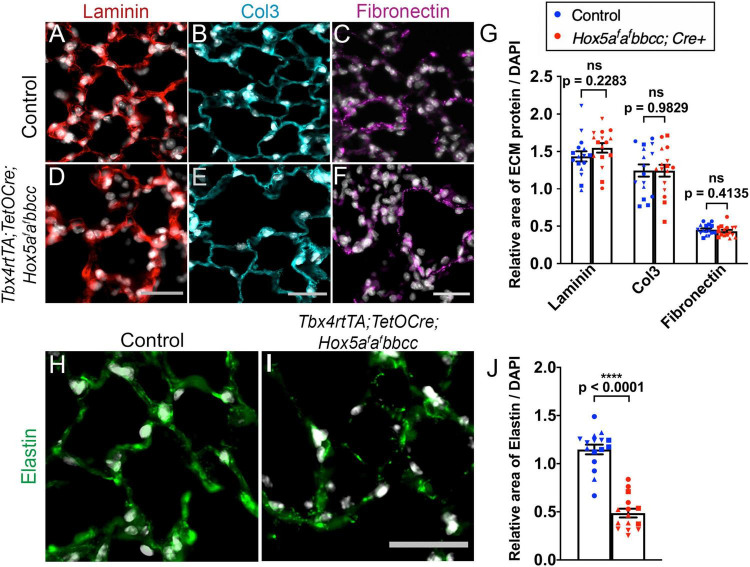
The elastin network is disrupted in the *Hox5* adult, conditional mutant lungs. Lung paraffin sections from control and *Hox5* adult, conditional mutant mice stained with laminin (red, **A,D**), collagen3 (cyan, **B,E**), fibronectin (magenta, **C,F**) and elastin (green, **H,I**) with DAPI in gray in all. Quantifications of pixel intensity of laminin, collagen3, and fibronectin was normalized to pixel intensity of DAPI, and show no differences between controls and mutants **(G)**. The elastin network is disrupted, and the total elastin pixel intensity normalized to pixel intensity of DAPI per field image is significantly decreased in mutant lungs compared to control lungs **(J)**. Each shape represents an individual animal (ns, not significant). Scale bars: 25 μm. *P*-values and statistical significance (****P < 0.0001) were determined by an unpaired Student’s *t*-test.

We analyzed several key ECM components in *Hox5* adult, conditional mutant distal lungs administered Dox from 8 to 10 weeks. Immunohistochemical immunofluorescence (IHC-IF) analyses with subsequent ImageJ quantification of pixel intensity demonstrated ECM phenotypes strikingly similar to those previously described with postnatal deletion (Hrycaj et al., 2018a). We observed no changes in the basement membrane component laminin, interstitial collagen 3 or fibronectin in the *Hox5* adult conditional mutant lungs compared to wild-type lungs (Figures 4A–G). However, we observed a substantial decrease in elastin staining (∼42% of wild-type levels) and disruption of the integrity of the elastin network in *Hox5* adult conditional mutant lungs (Figures 4H–J). This phenotype was particularly surprising as it indicates rapid loss and/or destruction of the elastin matrix that was fully established normally at 8-weeks of age and is considered very stable (Shapiro et al., 1991). RT-qPCR shows no differences of elastin mRNA level between controls and mutants, which indicates the changes in elastin structural integrity are likely an indirect consequence of other disruptions in the distal lung (Supplementary Figure 2F).

### Increased Inflammatory Response and Elastase Expression in the *Hox5* Triple Conditional Mutant Lung 3 Days After Dox Induction

**FIGURE 5:**
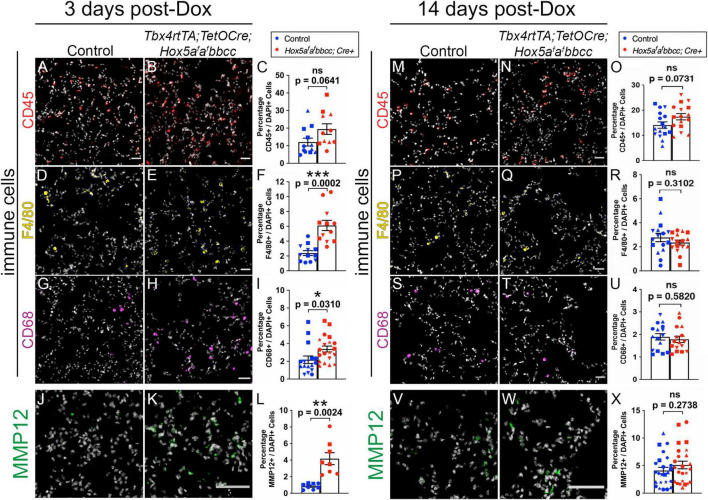
Increased inflammatory response of macrophages and MMP12 expression levels 3 days after *Hox5* deletion. Cryosections of control and mutant mice lungs (3 days post-Dox treatment initiated at 8 weeks of age) were stained with CD45 (red, **A,B**), F4/80 (yellow, **D,E**), CD68 (magenta, **G,H**), and MMP12 (green, **J,K**) with DAPI in gray. Quantifications of CD45- **(C)**, F4/80- **(F),** CD68- **(I)**, and MMP12- **(L)** positive cell numbers were normalized to DAPI-positive cell numbers and demonstrate significantly increased infiltration of macrophages and MMP12 staining levels in *Hox5* mutant distal lungs. No increase of lung leukocyte, macrophage populations or MMP12 expression levels was observed 14 days post-Dox treatment **(M–X)**. Each shape represents an individual animal (ns, not significant). Scale bars: 50 μm. *P*-values and statistical significance (*0.01 ≤ *P* < 0.05; **0.001 ≤ *P* < 0.01; ***0.0001 ≤ *P* < 0.001) were determined by an unpaired Student’s *t*-test.

Previous studies have demonstrated that leukocytes and macrophages secrete matrix metalloproteinases (MMPs), including neutrophil elastase (MMP12), that are capable of degrading the extracellular matrix during lung development and homeostasis (Gibbs et al., 1999; Grumelli et al., 2004; Zeng et al., 2014; Morrell et al., 2020). Further, in a study using *Hox5* compound null alleles (*Hoxa5*^+ ⁣/−^; *Hoxb5*^–/–^; *Hoxc5*^+ ⁣/−^), mice present with an increased Th2 cells response and exacerbated lung tissue pathology in asthma models (Ptaschinski et al., 2017). In order to determine whether an increase in either leukocytes or macrophages could account for the alveolar enlargement and the decrease in elastin in our *Hox5* conditional mutant lungs, we performed IHC-IF and quantifications for Cluster of differentiation 45 (CD45, also known as the leukocyte antigen) (Figures 5A–C and Supplementary Figures 3A,B), the macrophage marker F4/80 (Figures 5D–F and Supplementary Figures 3C,D), and Cluster of differentiation 68 (CD68), which labels tissue-resident macrophages (Figures 5G–I and Supplementary Figures 3E,F) immediately following *Hox5* deletion (3 days post-Dox treatment initiated at 8 weeks of age). We observed a significant increase in F4/80+ and CD68+ macrophages number in *Hox5* mutant distal lungs (Figures 5F,I), consistent with an increased inflammatory response following *Hox5* mesenchymal deletion.

Consistent with the influx of macrophages, we observed a significant increase of MMP12, a major elastase reported to be secreted by activated lung macrophages (McGarry Houghton, 2015), in *Hox5* conditional mutant lungs compared to controls 3 days after Doxycycline induction (Figures 5J–L). MMPs are shown to target both ECM components and adhesion receptors to alter cell behaviors in lung and other cancer cells (Yu and Stamenkovic, 2000; Rolli et al., 2003; Zeng et al., 2014). A previous study also suggests enhanced production of MMPs in *Hoxa5^–/–^* juvenile mice lungs (Mandeville et al., 2006). However, by 14 days after the initiation of deletion, there was no observable differences either in the number or location of leukocytes, macrophages (Figures 5M–U) or in the expression of MMP12 (Figures 5V–X and Supplementary Figure 4) between *Hox5* mutants and control lungs.

Together, these data are consistent with an acute inflammatory response in *Hox5 adult* mutant distal lungs after conditional *Hox5* deletion in the lung fibroblasts.

### *Hox5* Conditional Triple Mutant Fibroblasts Display Reduced Adhesion to Fibronectin *in vitro*

**FIGURE 6:**
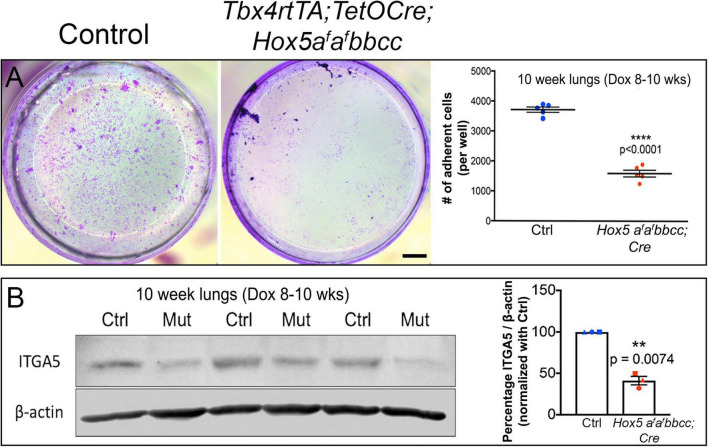
*Hox5* adult, mutant lung fibroblasts are less adherent in culture compared to controls. Lung fibroblasts were isolated, seeded on fibronectin-coated plates and incubated for 1 h prior to low-gravity centrifugation then fixation. Cells were stained with crystal violet, imaged, then counted. *Hox5* adult, conditional mutant stromal cells are significantly less adherent in this assay **(A)**. Western blotting of protein from right accessory lobes from *Hox5* adult, conditional mutant lungs show ∼50% lower ITGA5 levels compared to control lung tissues **(B)**. Each shape represents an individual animal. Scale bar: 10 mm. *P*-values and statistical significance (**0.001 ≤ *P* < 0.01; ****P < 0.0001) were determined by an unpaired Student’s *t*-test.

Our previous work examining lung fibroblasts from postnatal *Hox5* conditional mutants demonstrated that these cells exhibit reduced adhesion to fibronectin compared to controls (Hrycaj et al., 2018a). To examine whether *Hox5* plays a similar role in regulating adhesion of lung fibroblasts in response to deletion at adult stages (8–10 weeks of Dox-induced deletion), we cultured primary lung cells harvested from adult control and *Hox5* conditional mutant animals on fibronectin-coated plates. *In vitro* adhesion assays demonstrated that *Hox5* adult mutant fibroblasts exhibit a ∼60% decrease in their ability to adhere to fibronectin (Figure 6A). Consistent with what we reported after postnatal loss of *Hox5* function, there is a dramatic decrease in the protein expression of integrin α5, an important component of fibroblast adhesion, in mutants compared to controls (Figure 6B).

## Discussion

Although *Hox* genes were initially reported to be embryonic patterning factors, an increasing body of work has demonstrated that these genes play a role in the homeostasis, maintenance and repair in postnatal and adult tissues (Pineault et al., 2015; Rux and Wellik, 2017; Rux et al., 2017; Nova-Lampeti et al., 2018; Bradaschia-Correa et al., 2019; Song et al., 2020). Prior studies from our laboratory have demonstrated a requirement for *Hox5* in lung fibroblast adhesion and establishment of the elastin network during postnatal alveologenesis (Hrycaj et al., 2018a). In our present work, we extend these findings and show that loss of *Hox5* in the adult lung mesenchyme leads to a rapid expansion of the distal airspaces, apparently resulting from the degradation of the previously established elastin matrix responsible for maintaining alveolar structures in the distal lung. While other extracellular matrix components and distal lung cell types do not appear affected, we see a loss of adhesion in the fibroblasts of the distal lung and report an increase in macrophages and elastase (MMP12), which might contribute to the rapid change in alveolar structures.

The lung ECM, composed primarily of elastin, collagens, and proteoglycans, determines its mechanical properties. The elastin network specifically provides the elastic recoil necessary for exhalation and is reported to be stable, with a reported ^14^C half-life of ∼74 years or longer in humans (Shapiro et al., 1991). However, perturbations of the elastin network of the adult lung can cause destruction of the alveolar walls that result in emphysema, and mutations in components of the elastogenesis pathway, such as tropoelastin, fibrillins and fibulins are also associated with a predisposition to emphysema (Rodriguez-Revenga et al., 2004; Urban et al., 2005; Hersh et al., 2006; Hucthagowder et al., 2006). Additionally, elastin quantity is a marker of susceptibility to emphysema when the lung is challenged, as mice heterozygous for the elastin gene (*Eln*^+/–^) are more prone to develop emphysema after prolonged exposure to cigarette smoke (Shifren et al., 2007). This predisposition to emphysema was proposed to result from a lower availability of cross-linked elastin leading to degradation of the larger elastin fibers, and changes in the adult ECM leading to an increase in collagen that may ultimately prevent matrix remodeling by restricting cell movement (Shifren et al., 2007). In the adult *Hox5* conditional triple mutant lung, there is a rapid destruction of an already established elastin network. This could be mediated by an observed increase in hematopoietic-derived cell numbers, which leads to increased activated elastase (MMP12) in response to exposure of the matrix when fibroblasts lose adherence. The degradation of elastin would lead to an expansion of the distal airspaces reminiscent of that seen in emphysema (Grumelli et al., 2004; Shifren et al., 2007).

We previously showed that postnatal loss of *Hox5* in the lung mesenchyme leads to a decrease in the protein levels of the integrin α5β1 heterodimer (Hrycaj et al., 2018a), a phenotype which is recapitulated in the adult *Hox5* conditional triple mutant. Integrin α5β1 mediates the binding of lung fibroblasts to fibronectin (Watt and Hodivala, 1994; Liu et al., 2010, p. 1; Epstein Shochet et al., 2017), and is in turn regulated by the formation of focal adhesions (Cai et al., 2009). Overexpression of HoxA5 in EOMA cell lines has been shown to stabilize focal adhesions by increasing Akt expression (Arderiu et al., 2007), and other *Hox* genes also play a role in the regulation of adhesions *in vitro* (Jones et al., 1992). Intriguingly, increased MMP12 has been associated with a reduction of focal adhesions in patients with anti-alpha1 trypsin deficiency, resulting in an enhanced severity of emphysema (Baraldo et al., 2015). Overall, these data are consistent with an important role for *Hox5* genes in the regulation of lung fibroblast adhesion. Elucidation of the factors that regulate maintenance of the adult lung elastin matrix, including a better understanding of the role played by fibroblasts and by *Hox5* genes, will ultimately lead to better treatments for lung disease diseases related to lung mesenchymal behavior such as BPD, emphysema, and idiopathic pulmonary fibrosis (IPF).

## Data Availability Statement

The original contributions presented in the study are included in the article/Supplementary Material, further inquiries can be directed to the corresponding author.

## Ethics Statement

The animal study was reviewed and approved by Institutional Animal Care and Use Committee (IACUC) at the University of Michigan or Institutional Animal Care and Use Committee (IACUC) at the University of Wisconsin-Madison.

## Author Contributions

LM-S, DW, and M-HL designed the research. LM-S, M-HL, PvG, AR, and NL performed the research. M-HL, LM-S, AM, PvG, and DW analyzed the data. M-HL, LM-S, and DW wrote the manuscript. M-HL, PvG, AM, and DW revised the manuscript. All authors contributed to the article and approved the submitted version.

## Conflict of Interest

The authors declare that the research was conducted in the absence of any commercial or financial relationships that could be construed as a potential conflict of interest.

## Publisher’s Note

All claims expressed in this article are solely those of the authors and do not necessarily represent those of their affiliated organizations, or those of the publisher, the editors and the reviewers. Any product that may be evaluated in this article, or claim that may be made by its manufacturer, is not guaranteed or endorsed by the publisher.

## Supplementary Material

Supplementary Figure 1 Whole mount lung picture of *Hox5* conditional triple mutant lungs at 10 weeks of age (after Dox deletion from 8 to 10 weeks) show significantly expanded distal airspaces compared to controls (arrowheads, **A,B**). Scale bar: 100 μm.

Supplementary Figure 2 mRNA levels of *Sftpc* **(A)**, *T1*α **(B)**, *Pdgfr*α **(C)**, *Adrp* **(D)**, *Pecam1* **(E)** and *Elastin* **(F)** were normalized to β*-actin* and show no significant differences in control and *Hox5* conditional triple mutant lungs at 10 weeks of age (after Dox deletion from 8 to 10 weeks). Each shape represents an individual animal (ns, not significant). *P*-values were determined by an unpaired Student’s *t*-test.

Supplementary Figure 3 Low magnification depicting IHC-IF staining of CD45 (red, **A,B**), F4/80 (yellow, **C,D**) and CD68 (magenta, **E,F**) in 3 days post-Dox control and *Hox5* adult conditional mutant lungs. Scale bar: 1 mm.

Supplementary Figure 4 Protein levels of MMP12 appear low overall, but unchanged in 3-day **(A)** or 14-day **(B)** post-Dox treatment mutant lungs compared to controls demonstrated by western blots. The abundance of MMP12 protein was normalized to the total amount of protein in each lane. Each shape represents an individual animal (ns, not significant). *P*-values were determined by an unpaired Student’s *t*-test.
