# Supplementary figures and images for: The Lung Elastin Matrix Undergoes Rapid Degradation Upon Adult Loss of Hox5 Function
Source: Front Cell Dev Biol. 2021 Nov 26;9:767454. doi: 10.3389/fcell.2021.767454 (PMC8662386; doi:10.3389/fcell.2021.767454)

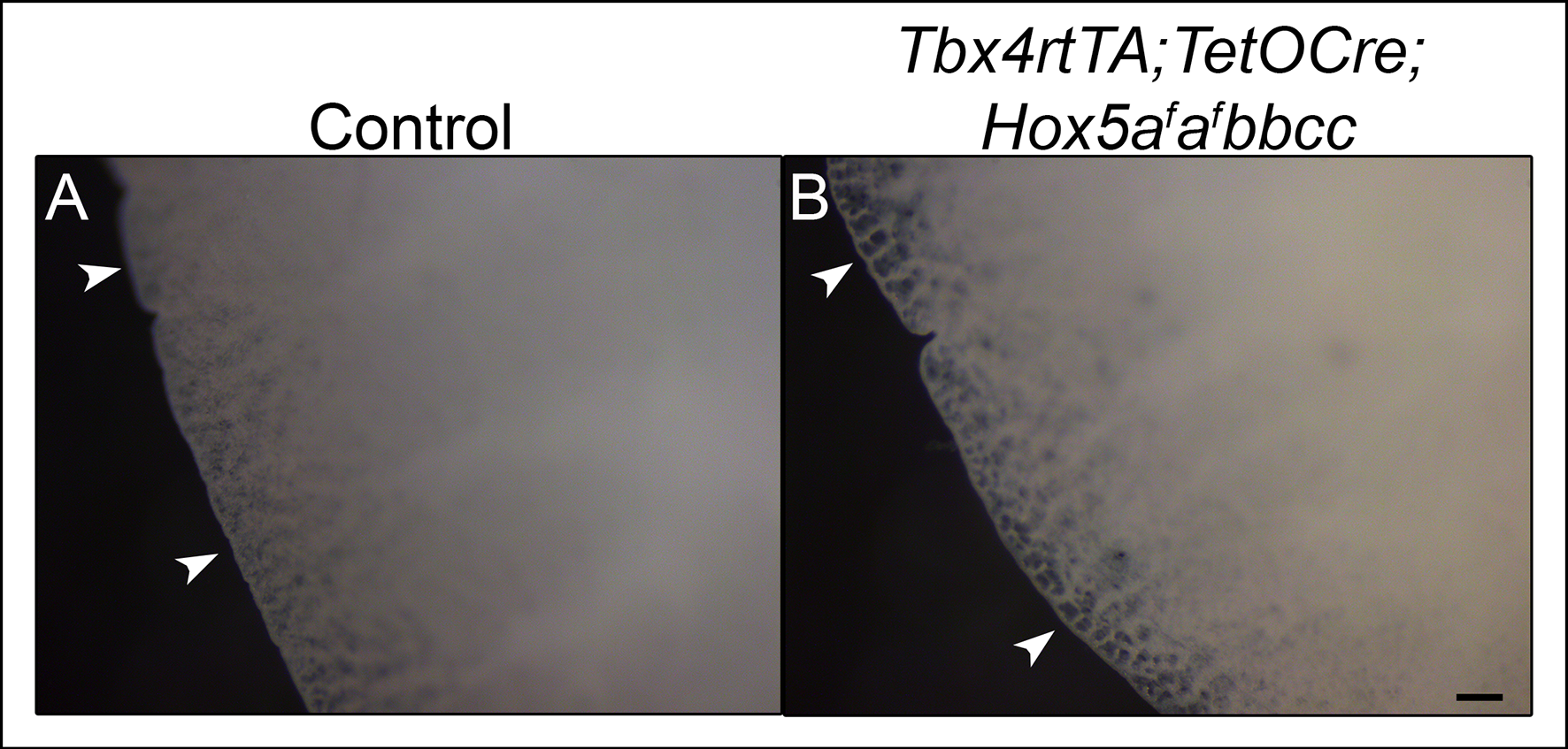

Supplement: Supplementary Figure 1 — Whole mount lung picture of Hox5 conditional triple mutant lungs at 10 weeks of age (after Dox deletion from 8 to 10 weeks) show significantly expanded distal airspaces compared to controls (arrowheads, A,B). Scale bar: 100 μm. [file Image_1.TIF]

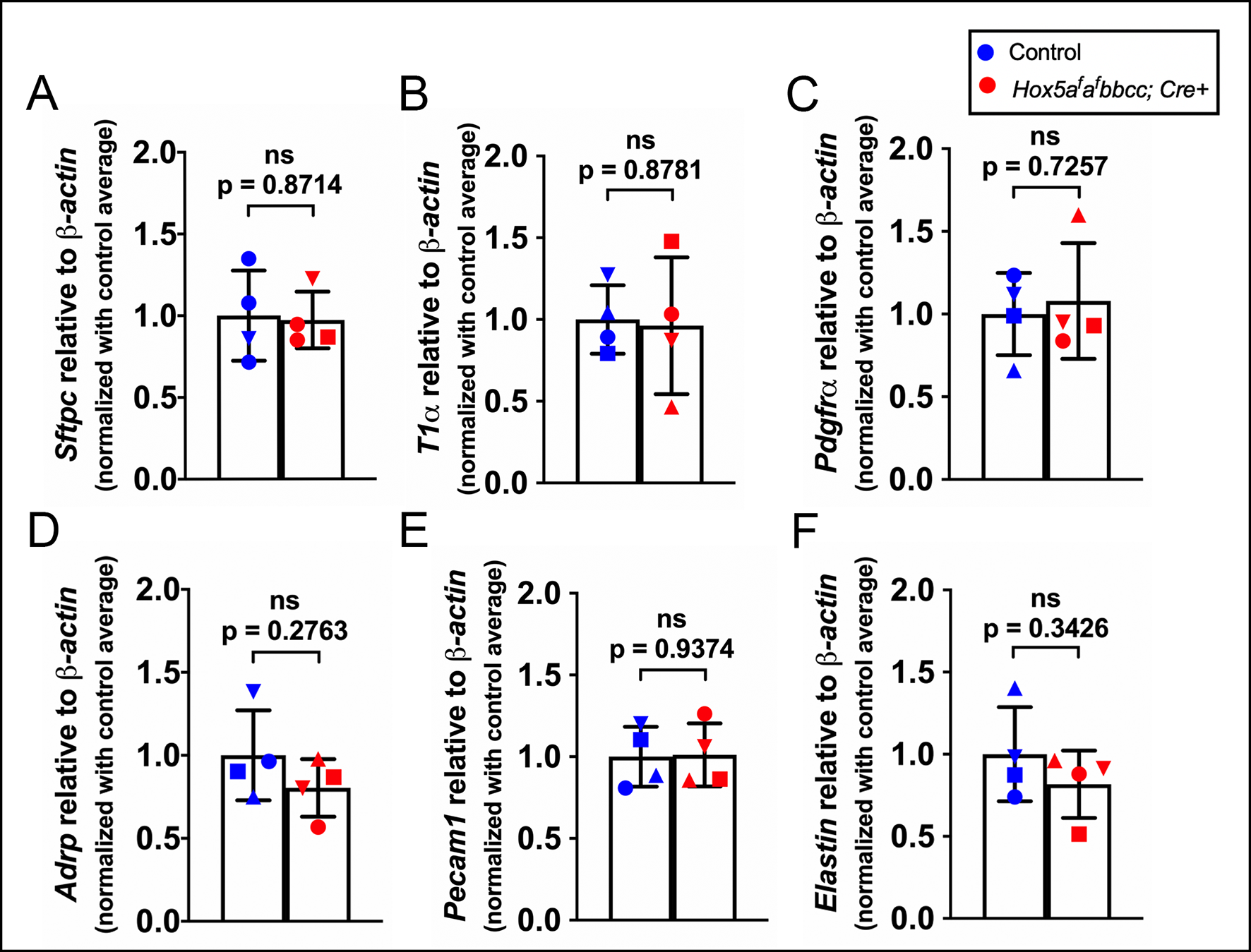

Supplement: Supplementary Figure 2 — mRNA levels of Sftpc (A), T1α (B), Pdgfrα (C), Adrp (D), Pecam1 (E) and Elastin (F) were normalized to β-actin and show no significant differences in control and Hox5 conditional triple mutant lungs at 10 weeks of age (after Dox deletion from 8 to 10 weeks). Each shape represents an individual animal (ns, not significant). P-values were determined by an unpaired Student’s t-test. [file Image_2.TIF]

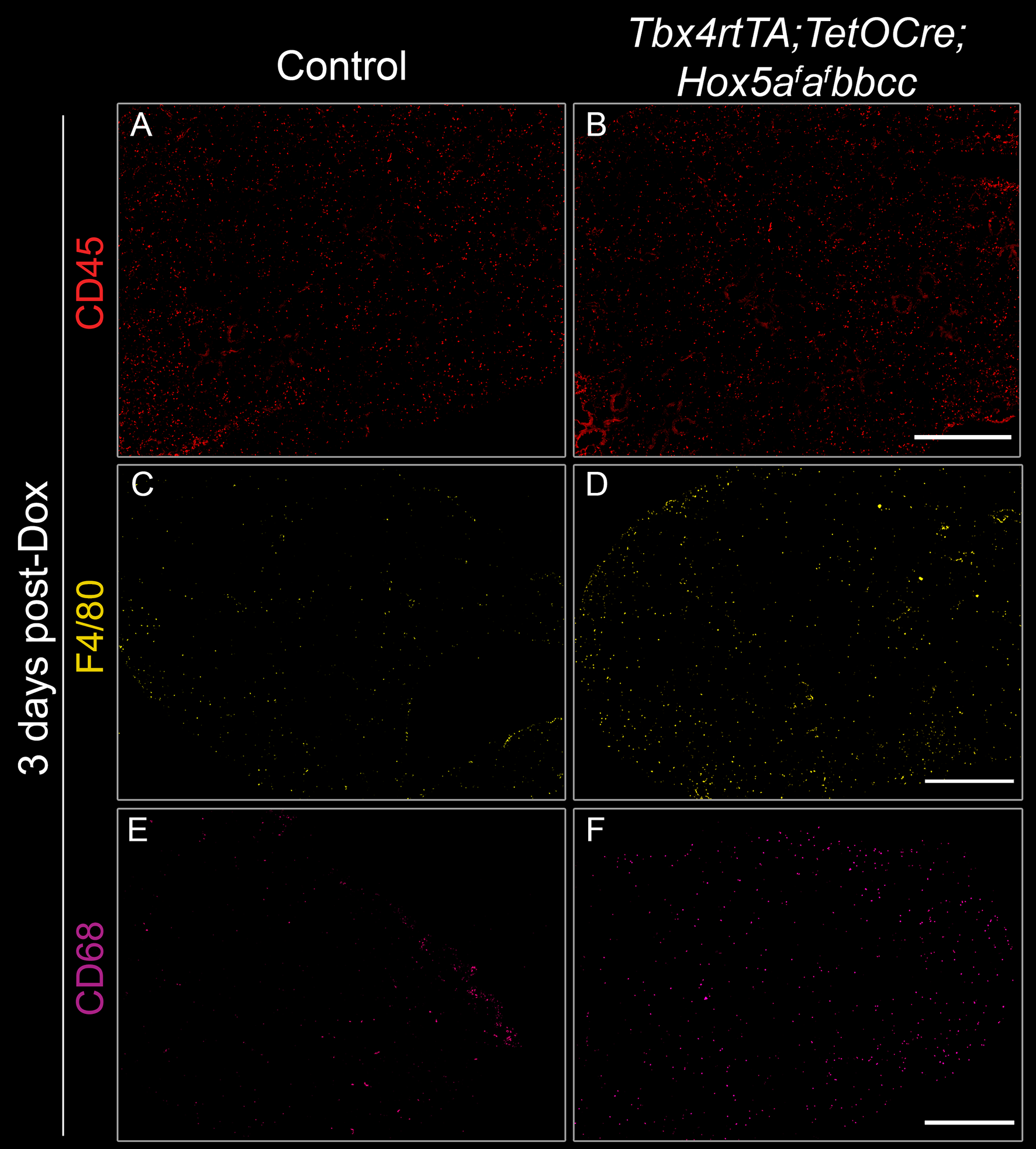

Supplement: Supplementary Figure 3 — Low magnification depicting IHC-IF staining of CD45 (red, A,B), F4/80 (yellow, C,D) and CD68 (magenta, E,F) in 3 days post-Dox control and Hox5 adult conditional mutant lungs. Scale bar: 1 mm. [file Image_3.TIF]

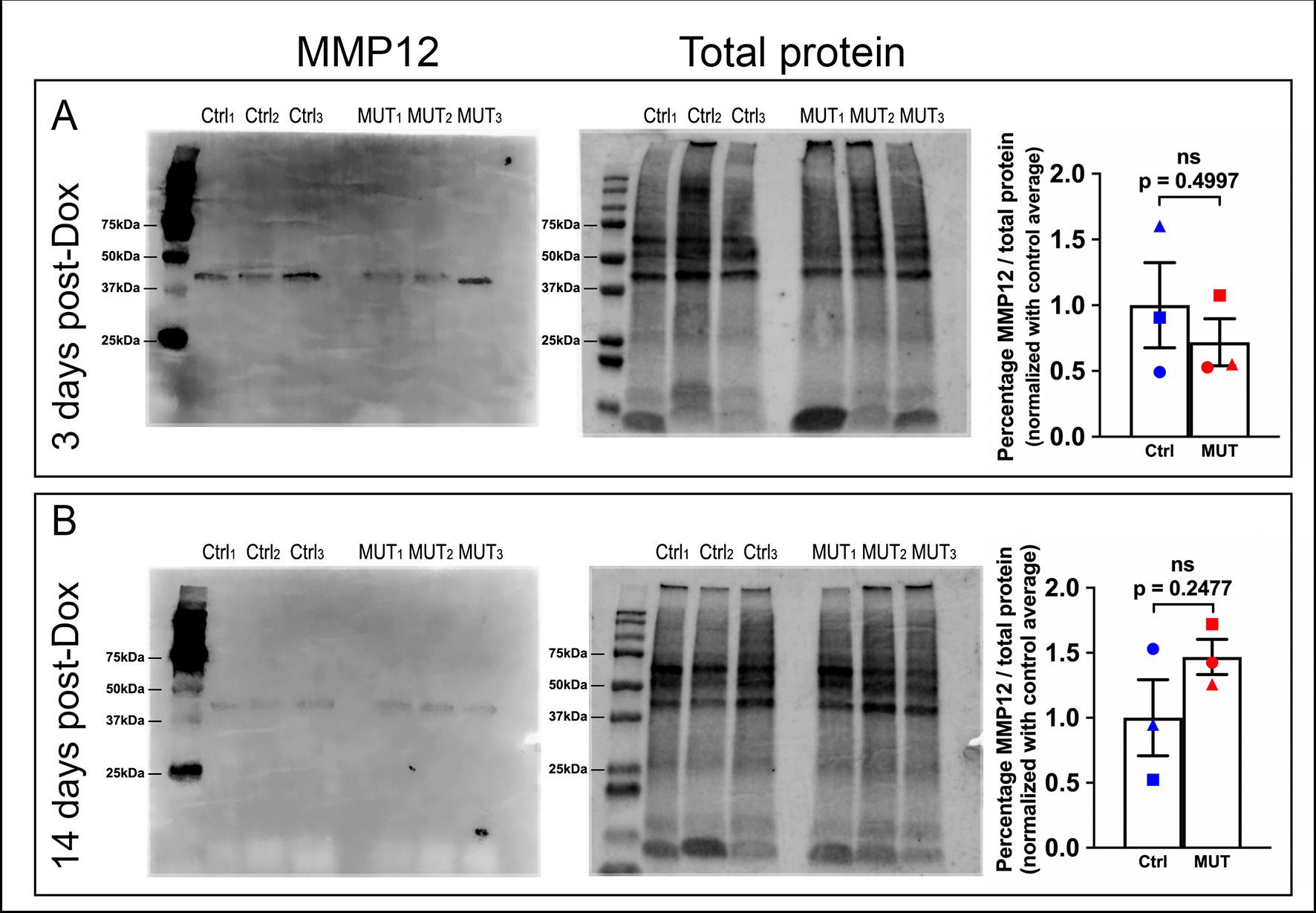

Supplement: Supplementary Figure 4 — Protein levels of MMP12 appear low overall, but unchanged in 3-day (A) or 14-day (B) post-Dox treatment mutant lungs compared to controls demonstrated by western blots. The abundance of MMP12 protein was normalized to the total amount of protein in each lane. Each shape represents an individual animal (ns, not significant). P-values were determined by an unpaired Student’s t-test. [file Image_4.TIF]
